# Protocol for metadata and image collection at diabetic foot ulcer clinics: enabling research in wound analytics and deep learning

**DOI:** 10.1186/s12938-024-01210-6

**Published:** 2024-01-29

**Authors:** Reza Basiri, Karim Manji, Philip M. LeLievre, John Toole, Faith Kim, Shehroz S. Khan, Milos R. Popovic

**Affiliations:** 1 Institute of Biomedical Engineering, University of Toronto, Toronto, Canada; 2 KITE Research Institute, Toronto Rehabilitation Institute – University Health Network, Toronto, Canada; 3 Zivot Limb Preservation Centre, Peter Lougheed Centre, Calgary, Canada; 4 Department of Surgery, Cumming School of Medicine, University of Calgary, Calgary, Canada; 5 Faculty of Kinesiology, University of Calgary, Calgary, Canada

**Keywords:** Diabetic foot ulcer, Deep learning, RGB, Depth, Thermal, Case report form

## Abstract

**Background:**

The escalating impact of diabetes and its complications, including diabetic foot ulcers (DFUs), presents global challenges in quality of life, economics, and resources, affecting around half a billion people. DFU healing is hindered by hyperglycemia-related issues and diverse diabetes-related physiological changes, necessitating ongoing personalized care. Artificial intelligence and clinical research strive to address these challenges by facilitating early detection and efficient treatments despite resource constraints. This study establishes a standardized framework for DFU data collection, introducing a dedicated case report form, a comprehensive dataset named Zivot with patient population clinical feature breakdowns and a baseline for DFU detection using this dataset and a UNet architecture.

**Results:**

Following this protocol, we created the Zivot dataset consisting of 269 patients with active DFUs, and about 3700 RGB images and corresponding thermal and depth maps for the DFUs. The effectiveness of collecting a consistent and clean dataset was demonstrated using a bounding box prediction deep learning network that was constructed with EfficientNet as the feature extractor and UNet architecture. The network was trained on the Zivot dataset, and the evaluation metrics showed promising values of 0.79 and 0.86 for F1-score and mAP segmentation metrics.

**Conclusions:**

This work and the Zivot database offer a foundation for further exploration of holistic and multimodal approaches to DFU research.

## Introduction

Approximately 540 million people have diabetes worldwide, and 90% have type 2 diabetes, primarily caused by socioeconomic, demographic, environmental, and genetic factors [1, 2]. Diabetes is a significant economic burden as its health expenditures are expected to reach trillions of dollars by 2030 and are anticipated to continue to rise [3]. Peripheral neuropathy is a common clinical symptom estimated to affect nearly 50% of diabetic patients, and of those patients, 25% are at greater risk of developing diabetic foot ulcers (DFUs) [4]. DFUs are full-thickness lesions below the ankle that breach the skin's dermis [5] and are a major complication of diabetes. DFUs are the leading cause of hospitalization and lower limb amputations (LLAs) [6]. DFUs can form due to various risk factors, such as trauma from high pressures in conjunction with neuropathy or peripheral arterial disease, foot deformities, calluses, and other comorbidities [7, 8]. DFUs are extremely resistant to healing, and DFUs treatment costs for both public and private payers have been established to be 9–13 billion dollars in the United States [9]. Due to the rising hospitalization of patients, mostly with LLAs, many facilities have adopted a multidisciplinary care team, which has shown to be of great benefit in treating major complications related to diabetes [10]. Hicks et al. [11] discovered that this would cost approximately $24,226 from presentation to healing, measuring approximately $2,412 per wound episode. The lack of available care space, human resources, time, and money to treat this ongoing rise in diabetes and DFUs demonstrates the need for exploring advanced approaches to manage and treat patients with DFUs.

Besides examining physical foot features, the three main DFU assessment methods are analysis of foot neuropathy, vascular circulation, and ankle-plantar pressures [12]. Once a patient has been screened and presents an ulcer, the current consensus surrounding DFU primary treatment involves debriding the wound, using offloading techniques, and providing local wound care [13]. Early detection and multidisciplinary care planning are key approaches to minimizing diabetes foot complications and hospitalization time. A wholesome evaluation of biomarkers and patient information would allow for more organized and personalized treatment plans. There are several biomarkers for DFU assessment and treatment, including the debridement approach [9, 14, 15], offloading methods [8], wound dressing, temperature, depth, odour, moisture, pain level, and healing phases [16, 17]. Although there have been general advancements in diabetes treatment, technologies to promote successful healing and recognizing biomarkers to identify non-healing patients and wound prognosis are still deficient [7]. To bridge the knowledge gap, aside from using molecular techniques to examine DFU biomarkers, researchers have begun to utilize artificial intelligence (AI) and advanced imaging tools to assess wounds’ severity and improve DFU management [7, 18]. As the incidence of infection in diabetics with ulcers is most concerning, the ability to skillfully detect infected or ischaemic DFUs can reduce the risk of hospitalization and, subsequently LLAs. AI approaches such as deep learning (DL) can be trained on large amounts of data (image or text) to provide personalized treatment recommendations [19]. Researchers have used DL and machine learning (ML) methods in DFU image classification, with better results and cost-effectiveness than traditional diagnostic methods [7, 20].

Research and development of such methods for DFU diagnosis and treatment have been limited for two reasons [21–25]: 1. absence of large holistic DFU datasets, and 2. incomparability of available datasets due to a wide variety of data collection methods. For example, whilst regular red–green–blue (RGB) images [26] are important in classifying DFUs, other factors such as temperature, moisture, odour, pain, wound onset, age, sex, and gender are essential in evaluating the wound condition [27, 28]. To this end, we created a comprehensive and large DFU dataset using our proposed data collection protocol. We called this DFU dataset, the Zivot dataset, to honour the hard work and assistance of Zivot Limb Preservation Centre clinicians, nurses and staff in gathering this database. Our main contributions are

- We created a novel, comprehensive and large DFU multimodal dataset using our proposed data collection protocol.
- Our data collection protocol was meticulously developed with expert clinical and research insights to be adopted as a gold standard for future DFU data collection.
- We showed the benefits of this dataset in a conventional DFU-related DL method.

### Protocol background

DL and ML techniques categorize DFUs automatically by employing extraction systems responsive to selective morphological features, shapes, sizes, and colours [20]. To accurately classify DFUs, DL uses deep neural networks, most commonly convolutional neural networks (CNN), that can efficiently extract informative features for image classification tasks [29]. To optimize the efficiency of DL and ML technologies, collecting demographic data such as age, sex, illness and DFU history, prior alcohol and smoking usage, wound characteristics, as well as comorbidities through case report forms (CRF), can aid in diagnosing underlying infection and lead to AI algorithms that successfully predict hard-to-heal DFUs [30, 31]. Other computerized solutions for DFU diagnosis, such as depth cameras, RGB sensors, and thermometry, are advanced imaging tools and have proven effective in medical settings [26, 32, 33]. Depth cameras accurately establish wound depth and area with less risk of observer error, wasted time and materials, and transmission of pathogens to the lesion than rudimentary methods, such as using rulers or metal probes [34]. RGB and thermal cameras can distinguish wound characteristics and measure superficial skin temperature, which can capture early signs of inflammation and infection, reducing the risk of DFUs in patients [35–37].

To function appropriately, ML algorithms and DL networks require large collections of unique data, such as thermal and RGB images, to identify and classify wounds [36]. However, there is a lack of good-quality datasets [20, 22] and certain DL models have difficulty classifying the severity of pain or detailed aspects of wound complications. From our standpoint, the extreme detriments diabetes has on individuals, families, and the healthcare system deem the augmentation of valuable DFU data and the implementation of DL developments as necessary in accurately diagnosing DFUs and other diabetic complications. Thus, the scope of this protocol is to provide a comprehensive DFU dataset that includes relevant clinical information, RGB, depth, and thermal data to be used as a framework in clinical and DL research and development.

### Related work

The limited existing DFU datasets are either small in size or lack the inclusion of comprehensive attributes essential for DFU. The noteworthy datasets and their attributes are summarized in Table 1.

**Table 1:** DFU datasets

Databases	Size	Features			
		RGB	Depth	Thermal	CRF
Zivot dataset (ours)	3,700	✔	✔	✔	✔
DFUC2020 [26]	4,000	✔	×	×	×
Plantar Thermogram [33]	334	×	×	✔	×
Alzubaidi et al. [32]	754	✔	×	×	×

This table summarizes key attributes of significant DFU datasets. Yap et al. [26] introduced DFUC2020, providing 4000 RGB. Hernandez-Contreras et al. [33] presented Plantar Thermogram with 334 thermal images. Alzubaidi et al. [32] proposed 754 RGB images for enhanced DFU classification

In recent years, DL has proved to be an efficient method for predicting and managing DFUs. However, DL algorithms must be fed sufficient and accurate data to maximize performance. Some of the more notable DFU datasets in the research community, such as DFUC2020 [26], and DFU_QUTNet [32] have shown promising results in detecting DFUs using only RGB images. However, with the growing research on incorporating thermal and patient health information in observing wound prognosis and healing, utilizing various data collection methods in creating DL datasets can produce improved results [38]. Furthermore, Hernandez-Contreras et al.’s research has expanded the knowledge on the use of AI in analyzing thermal images and DFU risk; however, expanding data to incorporate RGB, thermal, depth, and CRF information can produce a more comprehensive and efficient dataset. Thus, a challenge that remains in the literature is the lack of current datasets that utilize a wide variety of data modalities. It has been noted that fusing thermal and RGB images has improved the accuracy of DL methods for DFU segmentation compared to only using one or the other [39]. In addition, including patient details such as age, sex, wound onset, ulceration history, as well as wound characteristics such as location, exudate, depth, odour, and pain has been proven critical in assessing wound status [27]. Therefore, combining RGB, depth, thermal, and CRF in a dataset is crucial in the development of a precise and robust DFU prediction. Our proposed dataset integrates these four aspects and can be used as an advanced resource in DL development as it overcomes the drawbacks of past methods.

Unlike smaller datasets, which might lack the extent and depth needed for a holistic understanding, the Zivot dataset's inclusion of RGB, thermal, depth, and clinical vital information forms a robust foundation for multimodal data analysis. This comprehensive data allow researchers to explore the intricate interplay between visual, thermal, and clinical factors, shedding light on both apparent and underlying correlations that contribute to DFU progression and healing. The dataset's size further distinguishes it, enabling statistically significant findings and the potential to uncover insights that smaller datasets might overlook.

## Results

### Dataset analyses

There were 270 participants in the study and Table 2 indicates the summary of the demographics and Table 3 indicates the frequency of participant responses to consent-related DFU CRF questions. In this dataset, 78% of participants were male, and 83% had Type 2 diabetes. Participants diagnosed with sensory peripheral neuropathy, cardiovascular conditions, and cancers accounted for 89%, 42% and 11%, respectively. Predominantly, 88% of participants had one active DFU at the time of visit. Smoking habits and alcohol consumption patterns were recorded, with 85% having a lower or no smoking frequency and 77% having lower or no alcohol consumption. On average, each participant remained in the study for three follow-up appointments.

**Table 2:** Participant characteristics

Attributes	Average	Median
Age	62.1 ± 11.9	62.0
Weight (kg)	96.7 ± 28.2	92.5
Height (cm)	176.7 ± 9.3	177.0
Number of active DFUs per participant	1.2 ± 0.4	1.0

Averages and medians for key participant characteristics in the Zivot dataset

**Table 3:** Participant characteristics and responses

Attributes	Response	Frequency (%)
Sex	Male	200 (78%)
	Female	56 (22%)
Diabetes	Type 1	41 (17%)
	Type 2	202 (83%)
Diagnosed with any cardiovascular conditions	Yes	106 (42%)
	No	148 (58%)
Diagnosed with any cancers	Yes	29 (11%)
	No	227 (89%)
Diagnosed with sensory peripheral neuropathy	Yes	219 (89%)
	No	28 (11%)
Number of DFUs per participant	1	225 (88%)
	2	25 (10%)
	3	4 (2%)

Consumption level	Smoking—a cigarette	Alcohol—a glass
	Frequency (%)	Frequency (%)
Less or none	218 (85%)	198 (77%)
1 in 1–4 weeks	1 (~ 0%)	43 (17%)
Daily	7 (3%)	9 (4%)
More than 1 per day	30 (12%)	7 (3%)

Participant responses to consent-related CRF questions, including frequencies in both numbers and percentages

Data statistics presented in the tables are from responses available and recorded. Not every CRF attribute for the participants was filled out due to absence of data, participant uncertainty in response, or human error at the time of form filling. One participant was withdrawn, and data discarded when it was discovered the participant’s diagnosis of diabetes was mistakenly reported.

**Fig. 1:**
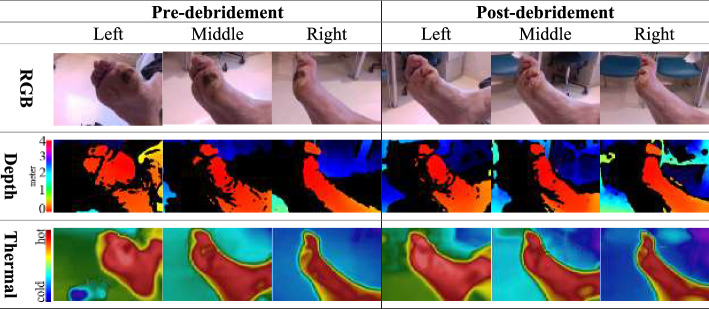
Illustration of image aspect of the Zivot dataset. The images were taken in two sessions, before and after debridement, with each session consisting of three different angles relative to the ulcer surface. The top row of the sample dataset shows the RGB images, whilst the next two rows illustrate the corresponding depth and thermal maps. In the maps, the colour red represents closer distances from the camera or higher temperatures in the depth and thermal maps, respectively. The colour blue indicates further distances from the camera or lower temperatures. Any pixel with no available value is shown in black, indicating missing data

As for the images, we collected 3686 RGB, 3680 depth and 3871 thermal images from the 269 participants over the 1 year (July 2022–June 2023), which included COVID-19-related slowdowns. The images were captured over 111 clinic days following our above-described image capture protocol. The different number of images for each modality is due to the presence of corrupted files, which were discovered during the post-processing of the dataset. The three image capture angles (left, middle and right) provided a natural augmentation, such as brightness, background alteration and perspective, as shown in Fig. 1. In addition, our approach to capture images before and after debridement stages provided both views of DFUs as they can be different in pre- and post-debridement, as shown in Fig. 1, top row. Images from depth and thermal cameras captured the same central area despite having different fields of view. As the cameras were placed in the box next to each other, DFUs appeared in the middle of images and approximately at the same position for both depth and thermal images. Thermal and depth cameras created regular RGB images and their respective thermal and depth maps. However, in some cases, maps were either missing areas without data (black) or corrupted due to artefacts such as frame drops or devices’ sensor malfunctions. Depth and thermal sensors demonstrated different sensitivity to lights, angles and distance, which made it difficult to capture the entire maps for every image capture consistently. Some areas of images appear black as the data are missing, as shown in Fig. 1 middle row. In the thermal images shown in the final row of Fig. 1, the spectrum of blue to green represents colder objects, which are predominantly located in the background. These images vividly depict the temperature variations in the DFU area before and after debridement, changes that are attributable to the improved blood circulation in the region.

### The Zivot dataset performance in deep learning

The results of the evaluations are summarized in Table 4 and Fig. 3. We demonstrated using the Zivot dataset with bounding box annotations and EfficientNet-UNet model, F1 score of 0.79 and mean average precision (mAP) of 0.86 are achieved for DFU detection in images.

**Table 4:** EfficientNet-UNet 3 fold cross-validation results

Evaluation metrics	F1 Score	mAP	Recall	IoU Score
	0.79	0.86	0.74	0.66

The network trained on Zivot dataset performed good in bounding box detection metrics

**Fig. 2:**
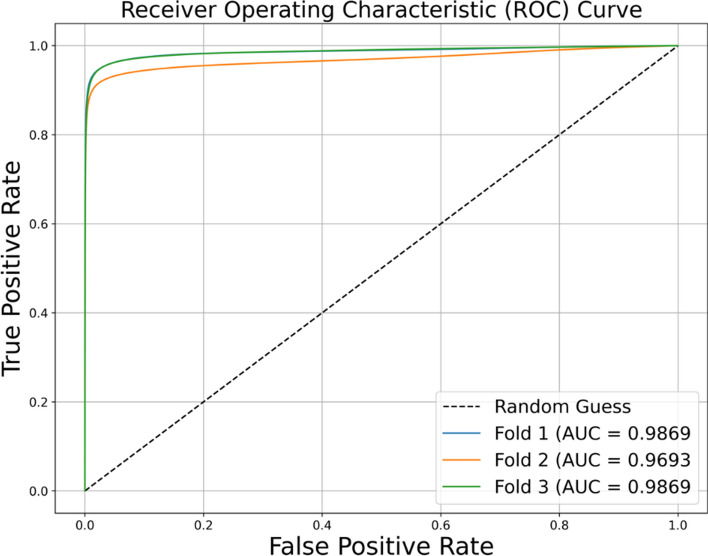
ROC curve analysis. ROC curve showcases the performance of the setup, indicating its ability to achieve high true-positive rates whilst maintaining low false-positive rates. The average AUC across the threefold is 0.98, with a standard deviation of 0.0082

**Fig. 3:**
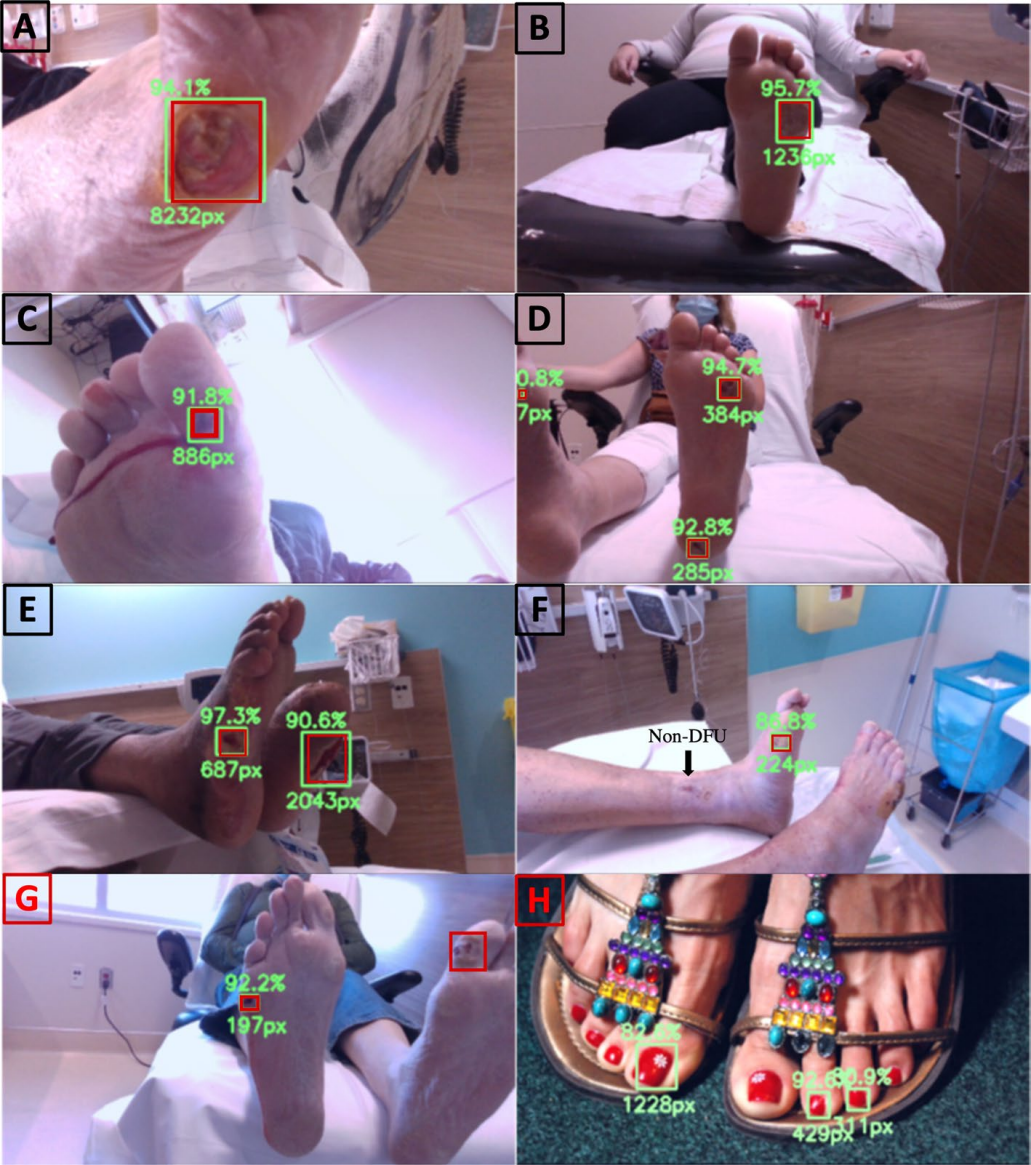
Versatile detection capabilities of the trained network. The red boxes represent the actual locations of wounds, whilst the green boxes show the predicted locations by the model. The following scenarios demonstrate the capabilities of the bounding box detection model. **A** Bounding box detection of a visible wound with surrounding box, confidence level, and bounding box area in pixels. **B** Early detection of a DFU, even with partial skin coverage, before debridement. **C** Successful DFU detection in challenging conditions like a bright background with a post-debridement ulcer. **D** detection of multiple ulcers in a single image. **E** Accurate detection of diverse skin tones, including dark skin. **F** Network specificity by correctly excluding a wound on the ankle from DFU detection. G and H illustrate failed cases where the second wound on the left foot is missed in G, and red nail polish is mistakenly marked as DFUs in H

In addition, the area under the curve (AUC) of the receiver operating characteristic (ROC) curve demonstrated that this setup achieves high true-positive and low false-positive rates with an average AUC of 0.98 for the threefold, as shown in Fig. 2. The bounding box detection of a clear wound is depicted in Fig. 3A. The box surrounds the detected DFU, the percentage value above the box indicates the confidence level of the network’s prediction, and the number below the box indicates the bounding box’s in pixels. The trained network detected DFUs in a wide variety of settings. For example, Fig. 3B illustrates the network's capability in detecting a DFU even before debridement when a thin layer of skin is covering the ulcer. Figure 3C and D shows the ability of the network to detect DFUs in conditions such as an abnormally bright background with a bloody post-debridement ulcer or multiple ulcers in one image, respectively. Figure 3E illustrates the insensitivity of the network to the participant’s skin tone where DFUs on dark skin feet were correctly detected. Figure 3F demonstrates the specificity of the network where a non-DFU wound on the ankle of the participant was rightfully not detected as a DFU. Finally, Fig. 3G and H illustrates negative results, where the second wound on the left foot is missed in G, and red nail polish is mistakenly marked as DFUs in H.

## Discussion

Our approach to DFU data collection was carefully designed to align with the demands of real clinical settings and the requisite efficiency. The CRF inquiries and image capture procedures were finely tuned to optimize acquiring essential, dependable data. For process consistency, common answers and options like "None" were included to pre-empt inadvertent errors and question skips. The questions were purposefully organized to mirror treatment steps, eliminating redundancy in clinician–patient interactions. In addition, because limited research has been done to evaluate the importance of different features in DFU healing phase classifications, we included most pertinent questions to extract as many key data elements as possible. As the DL-related research in DFU evolves, the CRF questions can be fine-tuned to be more specific. For example, a preliminary analysis of metadata within Tables 2 and 3 highlights potentially distinct features, such as patient weight and diagnosed cardiovascular conditions, which could hold significance in a DL network for predicting DFU healing phases, as opposed to the near-ubiquitous presence of sensory peripheral neuropathy for about 90% of the participants.

Beyond the optimized CRF, our work also introduces a noteworthy inclusion in the Zivot dataset: pre- and post-debridement images. This offers dual advantages. First, given the challenges patients face in self-performing debridement due to DFU location and mobility limitations, a DL detection network trained on the Zivot dataset can identify un-debrided DFUs, serving early detection and potential home-based supplementary treatment purposes. Second, as debridement is a vital DFU maintenance and treatment aspect, our trained network detected DFUs, including pre-debridement peri-ulcer regions and post-debridement ulcer areas. This functionality empowers clinicians to quantitatively assess and analyze debridement techniques and zones, enhancing their effectiveness. Figure 1 demonstrates the evaluation of debridement effects. Specifically, debridement is anticipated to improve blood circulation to the area of the DFU, thereby increasing the local temperature. This temperature change can be quantitatively assessed through thermal imaging. Prior to debridement, the wound is likely to exhibit a lower temperature due to the presence of necrotic tissue, which typically has poor blood supply. After debridement, a temperature rise is expected as a result of the acute inflammatory response, which is characterized by localized bleeding and inflammation. This response occurs following the removal of necrotic tissue, a phenomenon corroborated by the thermal images.

Although the presence or absence of sensory peripheral neuropathy was not a part of our recruitment criteria, we noted a higher incidence of diagnosed peripheral neuropathy compared to what is reported in the published literature. This underscores the notion that the diagnosis of this condition can encompass a broad spectrum of interpretations. Peripheral neuropathy can manifest either fully or partially in specific areas of the limb, and the diagnosis may vary depending on the scale and clinical judgement of healthcare providers. Consequently, we chose not to incorporate this condition as a definitive determining factor for our inclusion or exclusion criteria.

The DL application demonstrates a promising performance with a high F1 score of 0.79 and a mean mAP of 0.86 for detecting DFUs. Whilst it necessitates further analysis and comparison, these metrics surpass the previously established results in the DFU2020 dataset competition [26], which reported an F1 score of 0.74 and a mAP of 0.69. It is worth noting that the two datasets employed distinct protocols and camera systems for image capture, making it challenging to directly compare the performance of the Zivot dataset and the previously established metrics. The absence of a standardized protocol for data collection has constrained efforts to compare research findings and foster collaborative efforts in this field. By adopting the Zivot dataset protocol, we can expand and unify research and development efforts in DFUs. Figure 3, in particular, demonstrates a summary of our proposed detection model capabilities. The model predicts the location of wounds using green boxes, whilst the groundtruths are represented by red boxes. Even with partial skin coverage, an early detection of a DFU is made before debridement. Successful DFU detection is achieved even in challenging conditions like a bright background with a post-debridement ulcer. Multiple ulcers in a single image are detected. The detection of diverse skin tones, including dark skin, is accurate. The network is specific enough to exclude a wound on the ankle from DFU detection. Failed cases are illustrated in G and H where the second wound on the left foot is missed in G, and red nail polish is mistakenly marked as DFUs in H. To enhance the model's performance on failed cases, we suggest incorporating more healthy foot examples that exhibit similar wound features, such as red nail polishing. This additional augmentation and inclusion can help improve the model's accuracy and reliability.

## Limitations and challenges

The study encountered a key hurdle in the form of missing clinical and image data. Opting for paper-based CRFs aligned with the clinic's data system posed added complexities, including difficulty deciphering markings, incomplete CRF entries due to question skipping, record management challenges, and extensive paper handling. Transitioning to a digital CRF on tablets could alleviate these issues. Furthermore, some depth and thermal maps featured zones with uncollectible sensor data due to frame drops, suboptimal lighting, and sensor distance disparities. To mitigate data gaps in the maps, extra attention was given to ensuring data presence around the DFU site for both modalities during image capture. It is believed that depth and thermal cameras with more similar configurations and requirements could potentially be more suitable for this purpose.

Although the dataset offers valuable depth and thermal information, it has certain limitations when capturing fine-grained tissue characteristics depicted in these maps. Both cameras are specifically engineered for capturing macroscopic images, providing a broad field of view. The camera setup was intentionally designed to facilitate image capture at home for patients with restricted mobility and limited handholding capability. However, for studies necessitating microscopic tissue analysis and precise, well-lit, zoomed-in images, this dataset may offer restricted utility. As depth and thermal imaging technologies advance, the camera setup can be adapted to cater to a broader spectrum of applications and research studies.

## Conclusions and future work

This study introduces a comprehensive framework for collecting DFU data and facilitating advanced research in DFU analytics and DL. The protocol incorporates RGB, depth, and thermal images, along with crucial DFU attributes in the CRF. Employing this protocol, we successfully curated the extensive Zivot DFU dataset over a year, comprising about 3700 images from 269 participants and presented a summary of clinical and demographical features for this patient population. In addition, through training the selected EfficientNetb3-UNet DL network on this dataset, high-performance metrics were achieved, with an F1-score of 0.79 and mAP of 0.86. These outcomes establish a baseline for DFU detection using this dataset and underscore the protocol's and dataset's significance in advancing DL-based DFU research and development. Given the Zivot dataset's holistic nature, it offers ample potential for future multimodal DL advancements to harness its information richness fully.

This dataset opens new avenues to understand the healing and develop personalized treatment strategies for the patients. We showed the performance on RGB DFU images; in future, we will perform the same with depth and thermal cameras and their combinations to understand if multimodal images help in localizing the wounds more accurately. We will evaluate other DL segmentation and object detection models, including EfficientDet [40], YOLO [41], and Segment Anything [42], to perform a comprehensive analysis. We are also developing generative DL approaches using stable diffusion networks [43, 44] to create new wound images that can be used for data augmentation and reveal new artefacts that may be clinically relevant. We will develop a web application to identify real and generated DFU images (from different camera modalities) and seek clinical opinions on their accuracy. This dataset can also be used as a pre-trained model to fine-tune on other DFU datasets, such as DFU2020 and test on new or existing DFU datasets. However, translating models trained on the Zivot dataset to other datasets may induce dataset bias due to differing protocols, instruments and labelling [45]. New data bias mitigating strategies will need to be developed to maximize the utility of existing datasets.

## Methods

As this was a multicentre effort, The Conjoint Health Research Ethics Board of the University of Calgary (#21-1052) and the Research Ethics Board of the University Health Network (#21-5352) granted ethical approval for researchers conducting clinical research. All DFU patients were recruited from Zivot Limb Preservation Centre located in Peter Lougheed Hospital, Calgary, Alberta, Canada; permission to gather data was approved by Alberta Health Services (AHS) hospital authorities. Informed consent was taken from all the patients. To ensure confidentiality, no identifiable information about patients was kept in the research database, and any identifiable features, such as a clinic chart number, were replaced with a randomly generated code. Recognizable information such as age, sex, and weight remained with the research database but in separate storage from wound images, all password-protected on the hospitals’ servers and encrypted drives. Furthermore, the master list linking the randomized codes and any recognizable information was kept at AHS in a separate and secure location accessible only to the primary physician investigator.

### Protocol case report form

A detailed paper CRF was used for collecting preliminary patient data. The CRF was developed concisely and carefully in consultations with expert DFU clinicians, medical and DL researchers to maximize the usability and efficacy of the dataset. The medical history of participants was collected, if possible, at consent/baseline appointments as well as on follow-ups. A blank copy of the CRF used in the study is included in Appendix.

The CRF contains four sections: 1. Patient information, 2. Wound information, 3. Prescribed treatment plan, and 4. Healing phase estimation. The first page of the CRF contains basic information that is collected once at the first consent appointment. This information includes demographic aspects, such as age, sex, height, weight, number of DFUs, and type of diabetes (1 or 2). Smoking, alcohol consumption habits, comorbidities, including diagnosed cardiovascular conditions, cancer, and sensory peripheral neuropathy, as well as foot arch and toe characteristics such as bunions, claw/hammer, Charcot arthropathy, pes planus (flat arch), and pes cavus (abnormally high arch) are also noted on the first CRF page. The second page of the CRF contains information specific to the wound (i.e., location, temperature at wound centre and peri-ulcer, ulcer frequency whether new or recurrent, late-onset, level of pain, pain type, wound tunnelling, exudate amount/appearance, wound odour, peri-ulcer condition and the prescribed dressing by the DFU clinician. Page three includes any deformities or abnormalities on the affected foot, such as hair loss, dry skin, fissure cracks, callus, thickened toenails, and fungal nails. Patients’ offloading methods, if any, include therapeutic footwear, scotch cast boot, removable cast, half-shoes-healing sandals, total-contact cast, and crutches–walker–wheelchairs, are also recorded. The last section of the CRF contains a segment specifically for the DFU expert clinician to diagnose the current healing phase for each wound to the best of their abilities and to provide a confidence level percentage for their classification only for research purposes.

To maintain consistency in the dataset, for many categories, suggested picks for the patient and clinicians are provided in the CRF. For the clinician questions, subcategories of tunnelling include none, minor, medium, and severe. Exudate amount can be denoted by no exudate or dry, scant/small or moist, and heavy or wet/saturated. Exudate appearances are divided into four subcategories—serous or clear yellow fluid without blood, pus or debris, haemoserous, bloody/bright red, or thick, cloudy, mustard yellow or tan. Odour includes two categories—1. No or faint odour, and 2. Unpleasant/offensive/putrid. The peri-ulcer condition involves the subgroups: none, erythema/redness, oedema/swelling, hyperkeratosis, pale, or macerated. And last, dressing used on the wound has the options of none, gauze, promogran, urgo, inadine, idosorb, collagenase, and others. For the patient questions, the subcategory of the pain level on the foot with the wound was classified using numbers 0–5, depicting no pain, slight, moderate, noticeable, painful, and unbearable, respectively. Patients were asked to describe their pain type and options in the following proposed wordings: frequent waking at night or needing to dangle limb for relief, calf pain, pinching/throbbing, phantom, or other.

Most importantly, the CRF contains a section specifically for clinicians to diagnose the current phase of healing for each wound. The three healing phases that wounds can be classified into are 1. Inflammatory, 2. Proliferative, and 3. Remodelling/strengthening, and each phase can be further split into three subsections. The subdivision of each phase is denoted with a minus (−), no symbol, or a plus (+) sign to accurately determine if the wound is in the latter, middle, or final stages of the phase. Last, a percentage confidence level of the clinician’s wound classification is required to provide an accurate level of assessment and to act as a guideline to monitor patients’ progress. Standard healing phase visual guides were provided with CRF to assist clinicians' evaluations.

### Camera setup

**Fig. 4:**
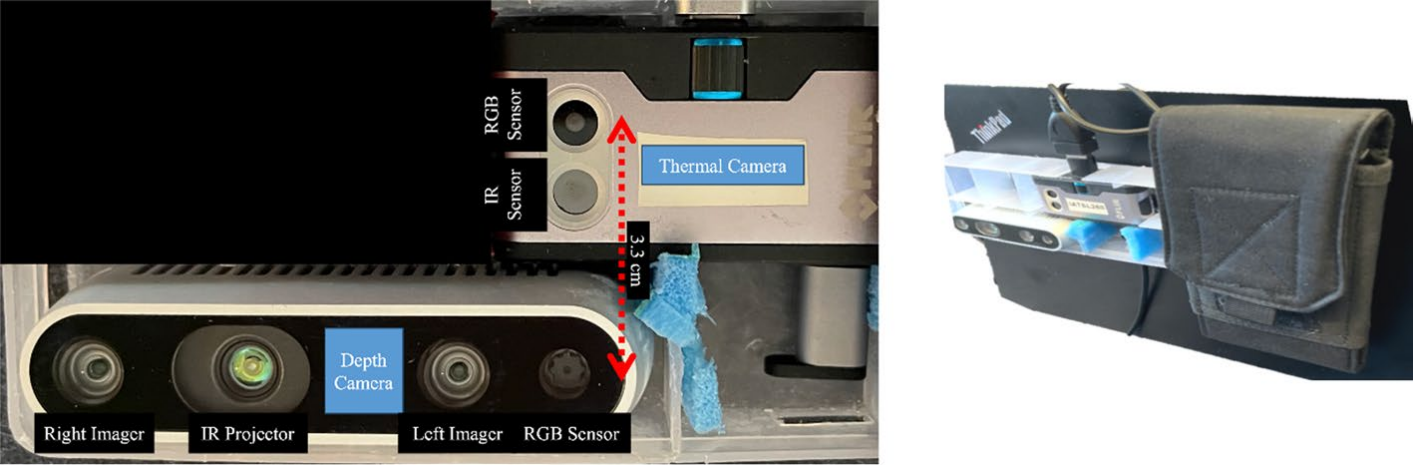
Camera setup and positioning. Left: the depth and thermal cameras were securely and adjacently positioned within a dedicated compartment. The RGB lenses were aligned and placed 3.3 cm from each other. The stereo and IR-generated depth, and thermal maps are superimposed on the RGB images for each camera. Right: the fixed apparatus was affixed to the rear of a laptop, enabling simultaneous image capture initiation from the laptop

Thermal and depth cameras were set up in a box for synchronized, consistent and approximately similar field-of-view capture of wound images, as shown in Fig. 4. Both cameras were placed close to each other so that the thermal and depth camera lens distances were measured at 3.3 cm. Cameras could not be placed on top of each other due to the heatsink requirements of the depth camera. For the thermal images, we used the FLIR ONE Gen 3 smartphone thermal infrared (IR) camera, and images were captured using the camera’s native Android mobile phone application with continuous calibration turned off in 1080 × 1440 dimensions. Each thermal image capture began with one camera calibration to the room lighting. For the RGB images and depth maps, we used the Intel RealSense D435i camera and RealSense viewer software. The stereo depth images were captured at 6 FPS, 16-bit and 1280 × 720 dimensions using the left and right imagers, as shown in Fig. 4. The RGB images were taken at 30 FPS, 8-bit and 1280 × 720 dimensions. Thermal and depth maps were projected onto the RGB images from thermal and depth cameras. The camera box containing the thermal camera, an Android smartphone, and the depth camera was attached to the back of a computer laptop, where the wound image capture was done with one click simultaneously from the laptop. Wound images were taken within a 1–2 feet distance between the surface of the wound and the front of the box where the camera lenses were located.

### Data collection

To select eligible participants for this dataset, we used the following inclusion and exclusion criteria. Inclusion patients must be individuals 18 years or older, have a valid diagnosis and confirmed diabetes record, and have a full-thickness DFU on the dorsal or plantar regions of the foot. For the exclusion criteria, patients unmotivated to participate in the study or unwilling to sign the consent form were excluded from the recruitment. Patients scheduled for amputation or undergoing additional interventions, such as surgery that would alter the natural DFU healing course, were also exempted. In addition, patients whose wounds were not experiencing any of the three healing phases were disqualified. Other exclusion criteria were patients having DFUs with gangrene or eschar, ongoing infection and drainage and thus cannot be debrided or exhibit extreme tunnelling or depth in a way that the primary clinician cannot visually inspect the depth of the wound and determine healing phases.

At the initial visit, all participants were screened by the podiatric surgeon based on the inclusion–exclusion criteria (see above) and the patient’s willingness to participate in this study, followed by consent form enrolment for the eligible participants. After the consent, participants were interviewed using the in-house developed CRF for past medical history, demographic details, and general foot and wound information.

At the recruitment appointment and pre-debridement, three pictures of the patient’s wound from left, middle, and right angles relative to the wound surface were taken. After the physician wound debridement, three additional post-debridement images in the same order were taken for a total of six images pre- and post-debridement. The temperature of the wound and the peri-ulcer skin were recorded using a Fora IR42 medical grade non-contact infrared thermometer digital thermometer with an accuracy of 0.2 Celsius. For foot temperature measurements, the thermometer mode was set to general body parts instead of the default forehead mode. If no debridement was required for a patient, only the three pre-debridement photos were taken. The physician then classified wound features and the healing phase of the DFU and provided a confidence percentage of the classification as outlined on the CRF. Images and forms were marked with timestamps to avoid misplacements. The image capture and CRF fillings were repeated for each patient at the follow-up appointments until the wound was classified as healed by the clinician or the participant withdrew from the study. All images were taken to exclude faces and identifiable information; however, the researchers took special considerations to blur any recognizable objects or aspects if included in the images.

### Deep learning evaluation on the Zivot dataset

The efficacy and value of this dataset were demonstrated in the wound detection aspect of DL. For these methods, only DFU RGB images of the dataset were used to establish a baseline with previously available performance metrics.

**Fig. 5:**
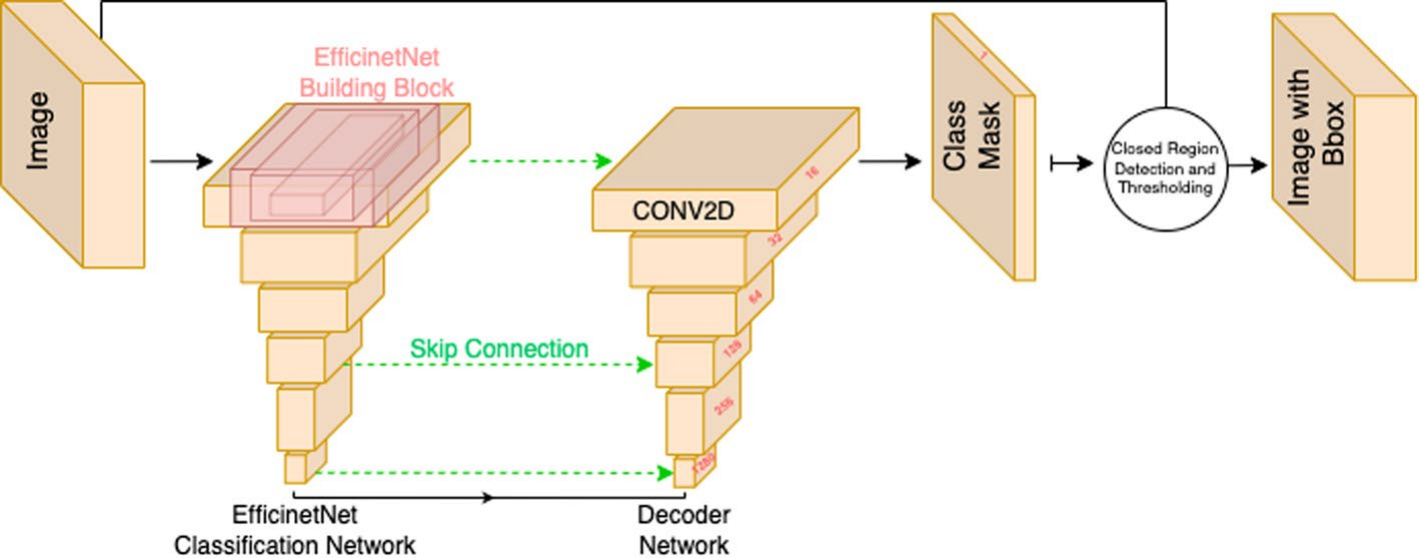
DL DFU detection architecture. A combination of EfficientNetb3 and UNet was used and trained with the Zivot dataset. This model has been shown to be a high-performing detection model for DFUs [46]

For object detection, we used UNet architecture with Efficientb3 feature extractor, Fig. 5, as it has been previously shown to be one of the highest performing combinations for the DFU detection [46]. For the detection dataset, 3686 (1280 × 720) RGB images were annotated with bounding boxes around each DFU by experts in the field. Network training was carried out using 480 × 480 random crops of the images, a decaying learning rate from 1e-03, a batch size of 70 and a cumulative loss function consisting of Dice, Jaccard, and Binary Focal losses. RGB colour channel distributions were normalized to the ImageNet dataset. Pixel level augmentations such as random contrast, blurring, brightness and transformation augmentations such as random flip and scale shift were implemented on the training dataset. To evaluate the benefit and application of the Zivot dataset in the DL domain, we performed a threefold cross-validation. The previously developed EfficientNet-Unet was trained three times on the entire dataset using a 0.67:0.33 split for training and testing datasets for each fold. F1 score, mAP, recall and intersection-over-union (IoU) metrics were used to evaluate the predicted bounding boxes around DFUs.

## Data Availability

Data cannot be shared publicly at this point because the release of data was not included in the ethics approval. We plan to facilitate the release of the dataset to selected research institutes upon request after ethics amendment approvals.

## Abbreviations

DFU: Diabetic foot ulcer
AI: Artificial intelligence
RGB: Red–green–blue
LLAs: Lower limb amputations
CNN: Convolutional neural networks
DL: Deep learning
ML: Machine learning
CRF: Case report form
mAP: Mean average precision
AHS: Alberta health services
AUC: Area under the curve
ROC: Receiver operating characteristic
IoU: Intersection over union

## References

[1] Sun H, Saeedi P, Karuranga S, Pinkepank M, Ogurtsova K, Duncan BB (2022). IDF diabetes atlas: global, regional and country-level diabetes prevalence estimates for 2021 and projections for 2045. Diabetes Res Clin Pract.

[2] Zheng Y, Ley SH, Hu FB (2018). Global aetiology and epidemiology of type 2 diabetes mellitus and its complications. Nat Rev Endocrinol.

[3] Garg SK, Rewers AH, Akturk HK (2018). Ever-increasing insulin-requiring patients globally. Diabetes Technol Ther.

[4] Hicks CW, Selvin E (2019). Epidemiology of peripheral neuropathy and lower extremity disease in diabetes. Curr Diab Rep.

[5] Schaper NC (2004). Diabetic foot ulcer classification system for research purposes: a progress report on criteria for including patients in research studies. Diabetes Metab Res Rev.

[6] Volmer-Thole M, Lobmann R (2016). Neuropathy and diabetic foot syndrome. Int J Mol Sci.

[7] Schmidt BM, Holmes CM, Najarian K, Gallagher K, Haus JM, Shadiow J (2022). On diabetic foot ulcer knowledge gaps, innovation, evaluation, prediction markers, and clinical needs. J Diabetes Complicat.

[8] Bus SA, Armstrong DG, Gooday C, Jarl G, Caravaggi C, Viswanathan V (2020). Guidelines on offloading foot ulcers in persons with diabetes (IWGDF 2019 update). Diabetes Metab Res Rev.

[9] Nube VL, Alison JA, Twigg SM (2021). Frequency of sharp wound debridement in the management of diabetes-related foot ulcers: exploring current practice. J Foot Ankle Res.

[10] Basiri R, Haverstock BD, Petrasek PF, Manji K (2021). Reduction in diabetes-related major amputation rates after implementation of a multidisciplinary model: an evaluation in Alberta, Canada. J Am Podiatr Med Assoc.

[11] Hicks CW, Canner JK, Karagozlu H, Mathioudakis N, Sherman RL, Black JH (2019). Quantifying the costs and profitability of care for diabetic foot ulcers treated in a multidisciplinary setting. J Vasc Surg.

[12] Bharara M, Schoess J, Armstrong DG (2012). Coming events cast their shadows before: detecting inflammation in the acute diabetic foot and the foot in remission. Diabetes Metab Res Rev.

[13] Core MAD, Ahn J, Lewis RB, Raspovic KM, Lalli TAJ, Wukich DK (2018). The evaluation and treatment of diabetic foot ulcers and diabetic foot infections. Foot Ankle Orthop.

[14] Kirshen C, Woo K, Ayello EA, Sibbald RG (2006). Debridement: a vital component of wound bed preparation. Adv Skin Wound Care.

[15] Edwards J, Stapley S, Edwards J (2010). Debridement of diabetic foot ulcers. Cochrane Database Syst Rev.

[16] Kavitha KV, Tiwari S, Purandare VB, Khedkar S, Bhosale SS, Unnikrishnan AG (2014). Choice of wound care in diabetic foot ulcer: a practical approach. World J Diabetes.

[17] Junker JPE, Kamel RA, Caterson EJ, Eriksson E (2013). Clinical impact upon wound healing and inflammation in moist, wet, and dry environments. Adv Wound Care.

[18] Pappachan JM, Cassidy B, Fernandez CJ, Chandrabalan V, Yap MH (2022). The role of artificial intelligence technology in the care of diabetic foot ulcers: the past, the present, and the future. World J Diabetes.

[19] Dilsizian SE, Siegel EL (2013). Artificial intelligence in medicine and cardiac imaging: harnessing big data and advanced computing to provide personalized medical diagnosis and treatment. Curr Cardiol Rep.

[20] Thotad PN, Bharamagoudar GR, Anami BS. Diabetic foot ulcer detection using deep learning approaches. Sensors Int 2023;4:100210. 10.1016/j.sintl.2022.100210.

[21] Goyal M, Reeves ND, Rajbhandari S, Ahmad N, Wang C, Yap MH (2020). Recognition of ischaemia and infection in diabetic foot ulcers: dataset and techniques. Comput Biol Med.

[22] Yogapriya J, Chandran V, Sumithra MG, Elakkiya B, Shamila Ebenezer A, Suresh Gnana Dhas C. Automated detection of infection in diabetic foot ulcer images using convolutional neural network. J Healthc Eng. 2022. 10.1155/2022/2349849. 10.1155/2022/2349849 PMC9007637 35432819

[23] Tulloch J, Zamani R, Akrami M (2020). Machine learning in the prevention, diagnosis and management of diabetic foot ulcers: a systematic review. IEEE Access.

[24] Anisuzzaman DM, Wang C, Rostami B, Gopalakrishnan S, Niezgoda J, Yu Z (2022). Image-based artificial intelligence in wound assessment: a systematic review. Adv Wound Care New Rochelle N.

[25] Kairys A, Pauliukiene R, Raudonis V, Ceponis J (2023). Towards home-based diabetic foot ulcer monitoring: a systematic review. Sensors.

[26] Yap MH, Hachiuma R, Alavi A, Brüngel R, Cassidy B, Goyal M (2021). Deep learning in diabetic foot ulcers detection: a comprehensive evaluation. Comput Biol Med.

[27] Ousey K, Chadwick P, Jawień A, Tariq G, Nair HKR, Lázaro-Martínez JL (2018). Identifying and treating foot ulcers in patients with diabetes: saving feet, legs and lives. J Wound Care.

[28] Dinh T, Veves A (2008). The influence of gender as a risk factor in diabetic foot ulceration. Wounds.

[29] Xu Y, Han K, Zhou Y, Wu J, Xie X, Xiang W. Classification of diabetic foot ulcers using class knowledge banks. Front Bioeng Biotechnol 2022;9:811028. 10.3389/fbioe.2021.811028. 10.3389/fbioe.2021.811028 PMC8918844 35295708

[30] Wang S, Xia C, Zheng Q, Wang A, Tan Q. Machine learning models for predicting the risk of hard-to-heal diabetic foot ulcers in a Chinese population. Diabetes, Metab Syndr Obes Targets Ther 2022;3347–59. 10.2147/DMSO.S383960. 10.2147/DMSO.S383960 PMC9628710 36341229

[31] Frescos N, Copnell B (2020). Podiatrists’ views of assessment and management of pain in diabetes-related foot ulcers: a focus group study. J Foot Ankle Res.

[32] Alzubaidi L, Fadhel MA, Oleiwi SR, Al-Shamma O, Zhang J (2020). DFU_QUTNet: diabetic foot ulcer classification using novel deep convolutional neural network. Multimed Tools Appl.

[33] Hernandez-Contreras DA, Peregrina-Barreto H, de Rangel-Magdaleno JJ, Renero-Carrillo FJ (2019). Plantar thermogram database for the study of diabetic foot complications. IEEE Access.

[34] Lasschuit JW, Featherston J, Tonks KT. Reliability of a three-dimensional wound camera and correlation with routine ruler measurement in diabetes-related foot ulceration. J Diabetes Sci Technol 2021;15(6):1361–7. 10.1177/1932296820974654. 10.1177/1932296820974654 PMC8655280 33243005

[35] Armstrong DG, Holtz-Neiderer K, Wendel C, Mohler MJ, Kimbriel HR, Lavery LA (2007). Skin temperature monitoring reduces the risk for diabetic foot ulceration in high-risk patients. Am J Med.

[36] Kaselimi M, Protopapadakis E, Doulamis A, Doulamis N (2022). A review of non-invasive sensors and artificial intelligence models for diabetic foot monitoring. Front Physiol.

[37] Lazo-Porras M, Bernabe-Ortiz A, Sacksteder KA, Gilman RH, Malaga G, Armstrong DG (2016). Implementation of foot thermometry plus mHealth to prevent diabetic foot ulcers: study protocol for a randomized controlled trial. Trials.

[38] Kim RB, Gryak J, Mishra A, Cui C, Soroushmehr SMR, Najarian K (2020). Utilization of smartphone and tablet camera photographs to predict healing of diabetes-related foot ulcers. Comput Biol Med.

[39] Bouallal D, Douzi H, Harba R (2022). Diabetic foot thermal image segmentation using Double Encoder-ResUnet (DE-ResUnet). J Med Eng Technol.

[40] Tan M, Pang R, Le QV. EfficientDet: scalable and efficient object detection, 2020; p. 10778–87. 10.1109/cvpr42600.2020.01079.

[41] Redmon J, Farhadi A. Yolov3: An incremental improvement. 2018 Apr 8. 10.48550/arXiv.1804.02767.

[42] Kirillov A, Mintun E, Ravi N, Mao H, Rolland C, Gustafson L, et al. Segment anything 2023. 10.48550/arxiv.2304.02643.

[43] Rombach R, Blattmann A, Lorenz D, Esser P, Ommer B. High-resolution image synthesis with latent diffusion models, Piscataway: The Institute of Electrical and Electronics Engineers, Inc. (IEEE); 2022. 10.1109/CVPR52688.2022.01042.

[44] Basiri R, Manji K, Harton F, Poonja A, Popovic MR, Khan SS. Synthesizing diabetic foot ulcer images with diffusion model. 2023. 10.48550/arXiv.2310.20140.

[45] Ashraf A, Khan S, Bhagwat N, Chakravarty M, Taati B. Learning to unlearn: building immunity to dataset bias in medical imaging studies, 2018. 10.48550/arxiv.1812.01716.

[46] Basiri R, Popovic MR, Khan SS. Domain-Specific Deep Learning Feature Extractor for Diabetic Foot Ulcer Detection. IEEE Int Conf Data Min Workshop ICDMW 2022; 2022-November:243–7. 10.1109/ICDMW58026.2022.00041.

